# IMMUNOREACT 0: Biopsy‐based immune biomarkers as predictors of response to neoadjuvant therapy for rectal cancer—A systematic review and meta‐analysis

**DOI:** 10.1002/cam4.6423

**Published:** 2023-08-03

**Authors:** Astghik Stepanyan, Matteo Fassan, Gaya Spolverato, Ignazio Castagliuolo, Melania Scarpa, Marco Scarpa, the IMMUNOREACT Study Group, Agostini Marco, Angriman Imerio, Bao Riccardo Quoc, Bardini Romeo, Becherucci Giulia, Bergamo Francesca, Bordignon Giovanni, Brignola Stefano, Brolese Marco, Businello Gianluca, Buzzi Gianluca, Campi Michela, Candioli Salvatore, Capelli Giulia, Cataldo Ivana, Cavallin Francesco, Cipollari Chiara, Chiminazzo Valentina, Da Lio Corrado, Dal Santo Luca, D’Angelo Antonella, De Simoni Ottavia, Dei Tos Angelo Paolo, Di Camillo Barbara, Di Cristofaro Loretta, Facci Luca, Franzato Boris, Gavagna Laura, Godina Mario, Guerrieri Mario, Guerriero Silvio, Guzzardo Vincenza, Kotsafti Andromachi, Laurino Licia, Marchegiani Francesco, Maretto Isacco, Massani Marco, Merenda Roberto, Mondi Isabella, Negro Silvia, Ortenzi Monica, Parini Dario, Pilati Pierluigi, Pirozzolo Giovanni, Porzionato Andrea, Portale Giuseppe, Pozza Anna, Pozza Giulia, Prando Daniela, Pucciarelli Salvatore, Recordare Alfonso, Ricagna Fabio, Rivella Giorgio, Romiti Chiara, Ruffolo Cesare, Saadeh Luca, Salmaso Beatrice, Salmaso Roberta, Scapinello Antonio, Scognamiglio Federico, Spolverato Ylenia Camilla, Stecca Tommaso, Tagliente Giovanni, Tomassi Monica, Tedeschi Umberto, Vignotto Chiara, Verdi Daunia, Zagonel Vittorina, Zizzo Maurizio

**Affiliations:** ^1^ UOC Chirurgia Generale 3 Azienda Ospedale‐Università Padova Padua Italy; ^2^ Department of Medicine DIMED University of Padua Padua Italy; ^3^ Veneto Institute of Oncology IOV‐IRCCS Padua Italy; ^4^ Department of Molecular Medicine DMM University of Padua Padua Italy; ^5^ Immunology and Molecular Oncology Diagnostics Unit Veneto Institute of Oncology IOV‐IRCCS Padua Italy

**Keywords:** lymphocytes, marker, neoadjuvant therapy, pathological complete response, rectal cancer

## Abstract

**Background:**

The main therapy for rectal cancer patients is neoadjuvant therapy (NT) followed by surgery. Immune biomarkers are emerging as potential predictors of the response to NT. We performed a meta‐analysis to estimate their predictive significance.

**Methods:**

A systematic literature search of PubMed, Ovid MEDLINE and EMBASE databases was performed to identify eligible studies. Studies on patients with rectal cancer undergoing NT in which the predictive significance of at least one of the immunological markers of interest was assessed by immunohistochemistry (IHC) in pretreatment biopsies were included.

**Results:**

Seventeen studies reporting sufficient data met the inclusion criteria for meta‐analysis. High levels of total CD3+, CD4+ and CD8+ tumor infiltrating lymphocytes (TILs), as well as stromal and intraepithelial CD8+ compartments, significantly predicted good pathological response to NT. Moreover, high levels of total (tumoral and immune cell expression) PD‐L1 resulted associated to a good pathological response. On the contrary, high levels of intraepithelial CD4+ TILs were correlated with poor pathological response. FoxP3+ TILs, tumoral PD‐L1 and CTLA‐4 were not correlated to the treatment response.

**Conclusion:**

This meta‐analysis indicated that high‐density TILs might be predictive biomarkers of pathological response in patients that underwent NT for rectal cancer.

## 1 INTRODUCTION

According to the guidelines of the National Comprehensive Cancer Network for Locally Advanced Rectal Cancer (RC), the current main treatment is neoadjuvant therapy (NT) followed by a total mesorectal excision.^1^ The benefits of NT include the reduction of tumor burden, the improvement of operative procedures and the prevention of local tumor recurrence compared to postoperative chemoradiotherapy treated patients.^2^ Pathological complete response (pCR) is defined as the absence of residual tumor cells in the resected specimen and lymph nodes at the time of surgery.^3^ It is used as surrogate endpoint to evaluate response to NT in RC.^4^ Given that only approximately 10%–30% of patients achieve pCR^5^ and NT can cause specific treatment‐associated toxicities,^6^ identification of reliable predictive biomarkers of response to NT represent a current important clinical challenge for RC patients management.

As NT induces cell death forms with immunogenic potential, immune cell composition in the tumor microenvironment might influence the response to NT. Indeed, cancer growth and progression are determined by the composition of the tumor microenvironment and the complex interactions between its components.^7^ In particular, the immune cell component of the tumor microenvironment has been shown to have both prognostic and predictive values.^8^, ^9^ The major subsets of tumor infiltrating lymphocytes (TILs) include CD3+, CD8+, CD4+, and FOXP3+ cells. The interaction between CD4+ T cell and antigen within MHC class II promotes production of cytokines which stimulate CD8+ T cell activation and proliferation.^10^ FoxP3+ T regulatory (Treg) cells in their turn can inhibit the activation and proliferation of CD4+ and CD8+ T cells, as well as cytokine production.^11^ A pro‐inflammatory tumor microenvironment characterized by cytotoxic CD8^+^ T cells and T helper 1‐oriented CD4^+^ T cells infiltration is usually associated with improved clinical outcomes; by contrast, the immunosuppressive functions of Treg and immune checkpoint molecules such as Programmed Death‐Ligand 1 (PD‐L1) appear to play a major role in promoting tumor immune escape.^12^ Indeed, the successful signaling of T‐cells is determined by TCR binding to major histocompatibility complex (MHC) and to costimulatory binding of CD80/CD86 to APC (antigen presenting cell) with CD28 molecules. Cytotoxic T lymphocyte antigen‐4 (CTLA‐4) is a competitive homolog of CD28 that prevents T‐cell activation. Similarly, the interaction between PD‐1 receptor and its ligand PD‐L1 and PD‐L2, that are expressed by both TILs and tumor cells such as CTLA‐4, can reduce proliferation, and survival of T‐cells.^13^, ^14^

Interestingly, accumulating evidence suggests that the extent and composition of infiltrating immune cells in RC might influence the response to NT. Indeed, several studies assessed the density and location of the major subtypes of tumor‐infiltrating lymphocytes (TIL) and immune checkpoint molecules to investigate their potential role as predictive biomarkers of treatment response and prognosis in RC patients undergoing NT.^15^, ^16^, ^17^, ^18^, ^19^, ^20^, ^21^ The evaluation of these predictive biomarkers could have a significant impact on the regimen choice of the preoperative combined therapy and the selection of patients for post‐operative treatments.

We thereby conducted a systematic review of studies assessing immune biomarkers by immunohistochemistry (IHC) in pretreatment biopsies of RC patients undergoing NT that used Tumor Regression Grade (TRG) as endpoint to evaluate the response to NT. In particular, we aimed to verify if TILs infiltration and immune checkpoint molecules expression might predict the response to NT.

## 2 METHODS

### 2.1 Study protocol

This systematic review was registered in the International Prospective Register of Systematic Reviews (PROSPERO) with an identification number CRD42022314065 on 2 March 2022.

### 2.2 Search strategy and screening

PubMed, Embase, and Ovid Medline databases were searched on the 21st of December 2021 with the following keywords: ((“rectal adenocarcinoma” OR “RC” OR “rectal neoplasm” OR “rectal carcinoma” OR “rectal tumo*”) AND (CD8 OR CTL OR CD4 OR CD3 OR FoxP3 OR Treg* OR cytotoxic T cell* OR helper T cell* OR “regulatory T cell*” OR CD80 OR CTLA4 OR CTLA‐4 OR PD1 OR PD‐1 OR PDL1 OR PD‐L1 OR PDL2 OR PD‐L2)). The search was restricted to original articles, English language and studies in humans. The search retrieved articles published from 1979 to 2021. Two authors (A.S. and Me.S.) made an inspection of the titles and abstracts of citations to identify relevant studies and obtain full texts. Two authors (A.S. and Me.S.) performed a review of all eligible manuscripts. Any contentious article was discussed with the other authors and an agreement was reached regarding articles for inclusion and exclusion.

### 2.3 Inclusion and exclusion criteria

Studies were eligible for inclusion if they met the following predefined criteria: (1) human studies on patients with RC undergoing NT followed by surgical resection; (2) Assessment of at least one of the immunological markers of interest (CD8, CD4, CD3, FoxP3, CD80, CTLA‐4, PD‐1, PD‐L1, PD‐L2) by immunohistochemistry (IHC) in pre‐treatment biopsies, (3) Evaluation of the pathological response in resected posttreatment surgical specimens by TRGs assigned to the high versus low level of biomarker. The exclusion criteria were as follows: (1) studies published in case reports or letters or comments forms or with no full‐text available; (2) Studies on patients who underwent exclusively short course radiotherapy with or without chemotherapy; (3) Studies providing only pCR/non pCR or no distinction between TRG grades, except if TRGs were categorized as good responders and poor responders.

### 2.4 Data extraction

The data were extracted into a spreadsheet. For each included study, the following data were extracted: first author's name, year of publication, journal, research center, study design (type, size, and recruitment period), patient clinical characteristics (age, gender, cancer stage), type of NT, specimen used for assessment (whether a biopsy or both biopsy and resected tissue), counting method (manual vs. digital), examined markers, markers location (intraepithelial TIL defined as lymphocytes in contact with tumor cells, stromal TIL defined as lymphocytes located in tumor stroma without direct contact with tumor cells), defined cutoff value, the definition of high versus low level of the markers, antibodies used (clones, manufacturers), time between end of radiotherapy and surgery, number of patients achieving pathologic complete response (pCR), TRG systems with derived definitions of good and poor responders. Studies were excluded from the meta‐analysis if corresponding authors of studies did not reply to email request for the missing data. The TRG data were extracted from studies that reported the definition of poor and good response according to the tumor regression grading. If the grades were presented individually, the good and poor responders were defined as presented in the Table S1. The meta‐analysis was performed using the total number of patients having good versus poor response which had high levels of biomarker.

### 2.5 Quality assessment

To assess the quality of the selected studies, a form originally developed by McShane et al.^22^ and Hayes et al.^23^ and further exploited by Mei et al.^24^ and Zhao et al.^25^ was adapted. Quality rating of 0–9 was based on reporting of inclusion and exclusion criteria, study design, patients and tumor characteristics, description of the method or assay used for marker assessment, description of the assay protocol, evaluation by more than one observer, study endpoints, TRG system specified, and quantitative evaluation of markers.

### 2.6 Statistical analysis

All meta‐analyses were performed using the Review Manager 5 software (RevMan 5, version 5.4.1). The meta‐analyses were conducted separately for each subgroup of markers of interest. The association of the level of markers and treatment response was analyzed with fixed‐effects model with 95% confidence interval (CI) assessing odd ratio (OR) using Mantel–Haenszel method. Heterogeneity was quantified by Chi‐squared (*X* ^2^) test and inconsistency index (I^2^) statistics.

## 3 RESULTS

### 3.1 Search results

**FIGURE 1:**
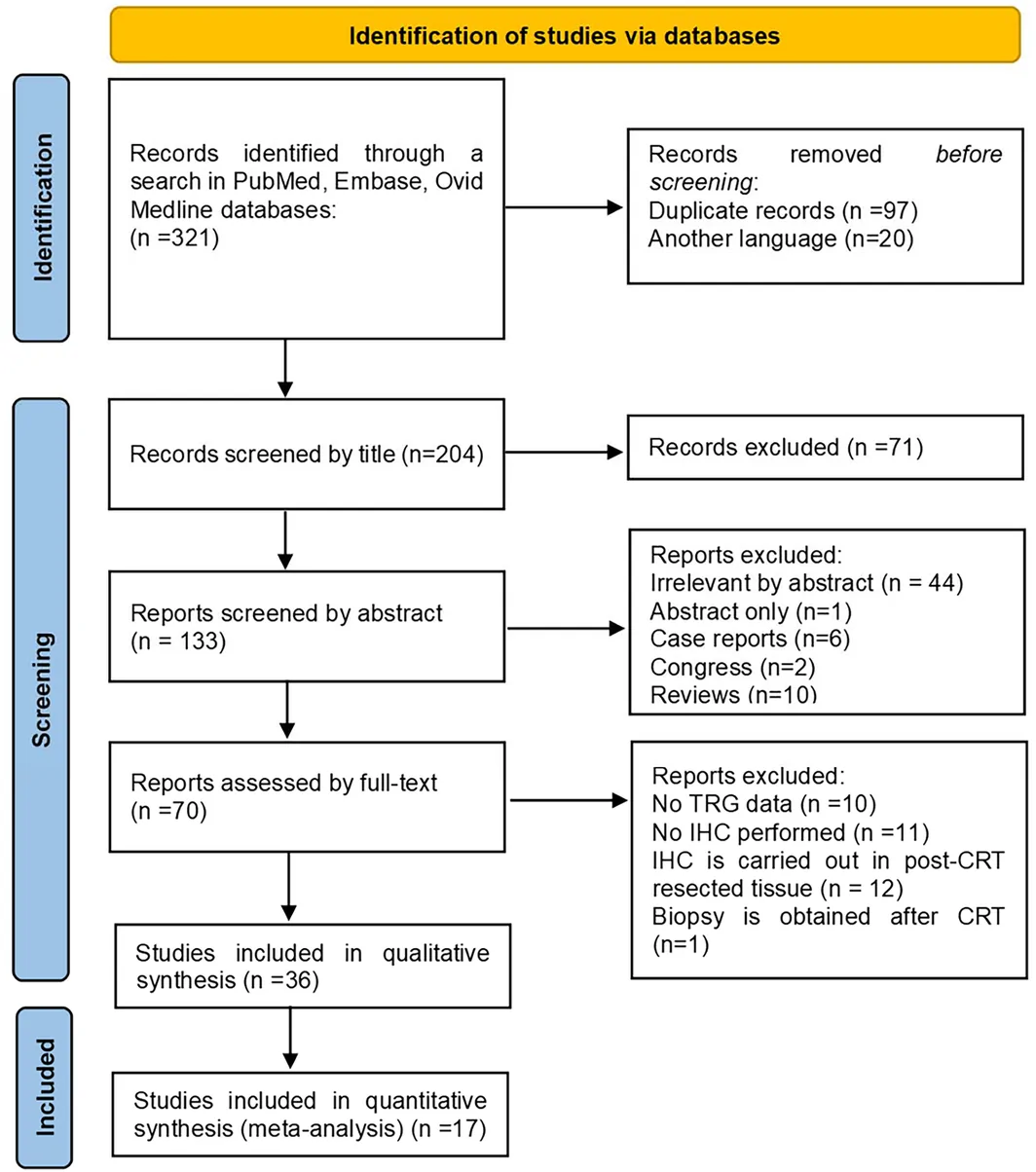
Prisma flow diagram of the search strategy.

The literature search retrieved a total of 321 unique study entries across the three databases. After exclusion of duplicates (*n* = 97) and non‐English language (*n* = 20), titles and abstracts were reviewed for 204 studies. After abstract review, 70 articles were identified for full manuscript review and 34 of these studies were excluded. The most common reasons for exclusion were the following: lack of TRG data (*n* = 10); no IHC performed for marker detection (*n* = 11); IHC performed on posttreatment specimen (*n* = 12). Thirty‐six articles were included in the final review, fulfilling all inclusion criteria, but 17 studies had insufficient data for meta‐analysis and two represented single studies of a particular marker. Therefore, 17 studies were included in the final meta‐analysis (Table 1). The detailed process of study selection is presented in Figure 1. There were no eligible studies with data for CD80 and PD‐L2 biomarkers. PD‐1 and stromal PD‐L1 data were found in single studies,^26^, ^27^ thus they were excluded from meta‐analysis.

**TABLE 1:** The main characteristics of the studies included in the meta‐analysis.

First author and published year	Research center	Study design	Study size	Lymphocyte subsets/markers of interest	Defined cutoff value	Definition of high versus low infiltration of lymphocytes in biopsy	Tumor regression grade system
Akiyoshi et al. 2019	Japan	single, prospective	275	CD8 (iTILs and sTILs)	Median density value cutoff: NR	CD8+ iTIL and CD8+ sTIL <cutoff/low, ≥cutoff/high	Dworak's TRG
Boustani et al. 2020	France	single, retrospective	73	PD‐L1, CD8*	Median rates of cells expressing PD‐L1 cutoff = 0.15	totPD‐L1 ≤ 0.15/low, > 0.15/high	Mandard's TRG
Chen et al. 2019	Taiwan	single, prospective	112	PD‐L1, CD8*	Semiquantitative, staining intensity (0–3+, Cutoff =2, 5% expression threshold	tPD‐L1 0‐1/low, 2‐3/high in more than 5% cells	CAP TRS
Chiang et al. 2019	Taiwan	single, retrospective	102	PD‐L1	Semiquantitative, staining intensity 0–3, Cutoff =2, 5% expression threshold	tPD‐L1 0‐1/low, 2‐3/high in more than 5% cells	Dworak's TRG
Farchoukh et al. 2021	USA	single, retrospective	117	CD8	ROC curve, cutoff = 157	totCD8 ≥157/high, low <157/high	CAP TRS
Huang et al. 2019	China	single, prospective	141	CD4, CD8 (iTILs and sTILs individually)	Cell density cutoff CD4+ iTIL = 1, CD4+ sTIL =41 iTILCD8 = 4.5, sTILCD8 = 42.5	CD4+ iTIL ≥ 1/high, <1/low; CD4+ sTIL ≥ 41/high, <41/low; CD8+ iTIL ≥4.5/high, <4.5/low; CD8+ sTIL ≥ 42.5/high, <42.5/low	AJCC 7/UICC
Lim et al. 2017	Korea	single, retrospective	123	PD‐L1, CD8*	semiquantitative, staining intensity, 0–3	tPD‐L1 <cutoff/low, ≥cutoff/high	Dworak's TRG
Lai et al. 2020	China	single, retrospective	134	CD4, CD8 (iTILs and sTILs separately)	Ordinal quantiles (Q1‐4) of lymphocyte density	CD4+ iTIL: Q1 = 0/mm2 (low), Q2 > 0/mm2 (high); CD8+ iTIL: Q1 + Q2 ≤ 5/mm2 (low), Q3 + Q4 > 5/mm2 (high); CD4+ sTIL: Q1 + Q2 ≤ 52/mm2 (low), Q3 + Q4 > 52/mm2 (high); CD8+ sTIL: Q1 + Q2 < =41/mm2 (low), Q3 + Q4 > 41/mm2 (high)	Dworak's TRG
Lim et al. 2014	Australia	single, retrospective	52	CD3, CD4, CD8	Median density value, cutoff CD3:102 CD4:31 CD8:70	totCD3:<102.5/low, ≥102.5/high; totCD4:<31/low, ≥31/high; totCD8:<70/low, ≥70/high	Mandard's TRG
Matsutani et al. 2018	Japan	single, retrospective	64	CD4, CD8, FOXP3	Median density value, cutoff: CD8 = 8, CD4 = 4.6, Foxp3 = 13.6	totCD8: <8/low, ≥8/high; totCD4: <4.6/low, ≥4.6/high; totFoxp3: <13.6/low, ≥13.6/high	JCCC8
McCoy et al. 2017	Australia	single, prospective	106	CD8, FOXP3, CD3	Median density value, cutoff: Foxp3 = 427, CD8 = 373, CD3 = 1104 (n = 93)	totFoxp3: <427/low, ≥ 427/high; totCD8: <373/low, 373/high; totCD3: <1104/low, 1104/high	Dworak's TRG
Moghani et al. 2020	Iran	single, retrospective	83	CD8	Cell density, High infiltration (> 11 cells/HPF), low infiltration (2–10 cells/HPF), no infiltration (0–1 cells/HPF).	CD8+ iTIL: low+no infiltration/LOW, high infiltration/HIGH	AJCC7/UICC
Sawada et al. 2021	Japan, US	multicenter, retrospective	267	CD8	Median density value, cutoff: NR	CD8+ iTIL <cutoff/low, ≥cutoff/high	Dworak's TRG
Shinto et al. (2) 2020	Japan	Multicenter, retrospective and prospective	144	CD8	Median density value, cutoff: NR	CD8+ iTIL <cutoff/low, ≥cutoff/high	Dworak's TRG
Teng et al. (1) 2015	China	single, retrospective	136	CD8, CD3	Mean value of the cell density, cutoff: NR	totCD3 and totCD8 <cutoff/low, ≥cutoff/high	Dworak's TRG
Teng et al. (2) 2015	China	single, retrospective	62	CD4, CD8 FoxP3, CTLA4, PD‐L1*	CTLA‐4: Scores for intensity were 0 (none)‐3 (strong). The final IHC score (H score) (1+ Intensity) x Percentage, Foxp3+: number of positive cells, cut off = median CD8 and CD4: % of positive cells, Cutoff = median	CTLA‐4: H score < 20/low, ≥20/high; FoxP3: <11/low, ≥11/high; totCD4 and totCD8: <cutoff/low, ≥cutoff/high	Dworak's TRG
Zhang et al. 2019	China	single, prospective	109	CD4, CD8 FOXP3, PD‐L1, CTLA4	Mean value of the cell density	totCD4: 16.35 ± 8.76% totCD8: 17.64 ± 7.74% totCTLA4: 6.34 ± 5.66% totPDL1: 9.28 ± 8.77% totFOXP3: NR	Dworak's TRG

Abbreviations: AJCC 7/UICC, american joint committee on cancer 7/union for international cancer control, CAP, college of american pathologists; JCCC, japanese classification of colorectal carcinoma; NR, not reported; NR, not reported; s/i TIL, stromal/intraepithelial tumor infiltrating lymphocytes, t, tumoral, TRG, tumor regression grade; tot, tumoral + immune cell expression of marker or intraepithelial + stromal TILs; *, not involved in meta‐analysis.

### 3.2 Characteristics of selected studies

The 17 studies selected for meta‐analysis were published between 2014 and 2021. They were carried out in Asia (*n* = 12), Australia (*n* = 2), Europe (*n* = 1), Middle East (*n* = 1) and USA (*n* = 2). There were 12 retrospective and six prospective cohort studies, set up as single (*n* = 16), and multicenter (*n* = 1) studies. The median study size was 105.5, with an interquartile range of 57. Thirty percent of studies included AJCC stage II, III cancers, 53% included stage I–III cancers, and 12% consisted of I–IV stage cancers, 5% of studies did not report the stage of cancer. All patients underwent NT, most of the patients were subjected to CRT. In all selected studies markers of interest were evaluated by IHC staining in pretreatment biopsies, with 65% of studies assessing more than one immune marker of interest. Sixty‐five percent performed immune marker IHC on whole sections, 35% on tissue microarrays. Automated cell counting was utilized in nine studies, while the remaining eight used manual assessment methods. Thirteen studies assessed the marker levels quantitatively based on different cutoff values for dichotomizing markers into groups of high versus low levels. Most studies used the median value, mean values or combined scores as cutoff. For the assessment of TRG, 10 studies used Dworak's system, two used Mandard's, two used AJCC 7/UICC, two used CAP TRS, one used JCCC8. Only 11 studies reported the time between end of therapy and surgery. The detailed characteristics of the studies are summarized in Table S2. The studies had a median quality score of six out of nine (range: 5–7); the quality of each study is presented in Table S3.

### 3.3 TIL as predictors of response to therapy

#### 3.3.1 CD3+ T lymphocyte subset

**FIGURE 2:**
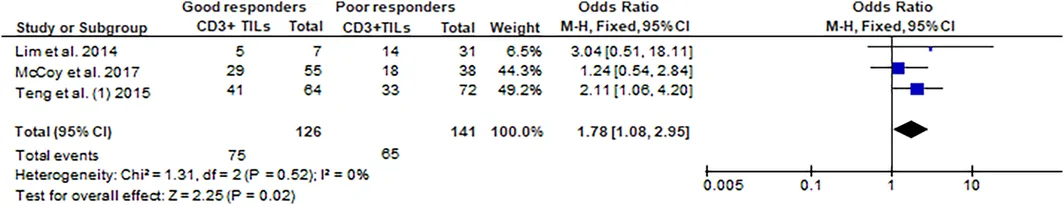
Forest plot representing the correlation between high level of CD3+ TILs and response to NT.

T lymphocytes are a basic component of the adaptive immune response characterized by expression of the cluster of differentiation (CD) cell surface marker CD3. The relationship between the presence of CD3+ T lymphocytes and response to NT was extracted from three studies.^17^, ^28^, ^29^ The results of the meta‐analysis demonstrated that high levels of CD3+ TILs in patients' biopsy specimens significantly predict good pathologic response (OR 1.78, 95% CI 1.08–2.95; participants = 267; I^2^ = 0%) (Figure 2).

#### 3.3.2 CD4+ T lymphocyte subset

**FIGURE 3:**
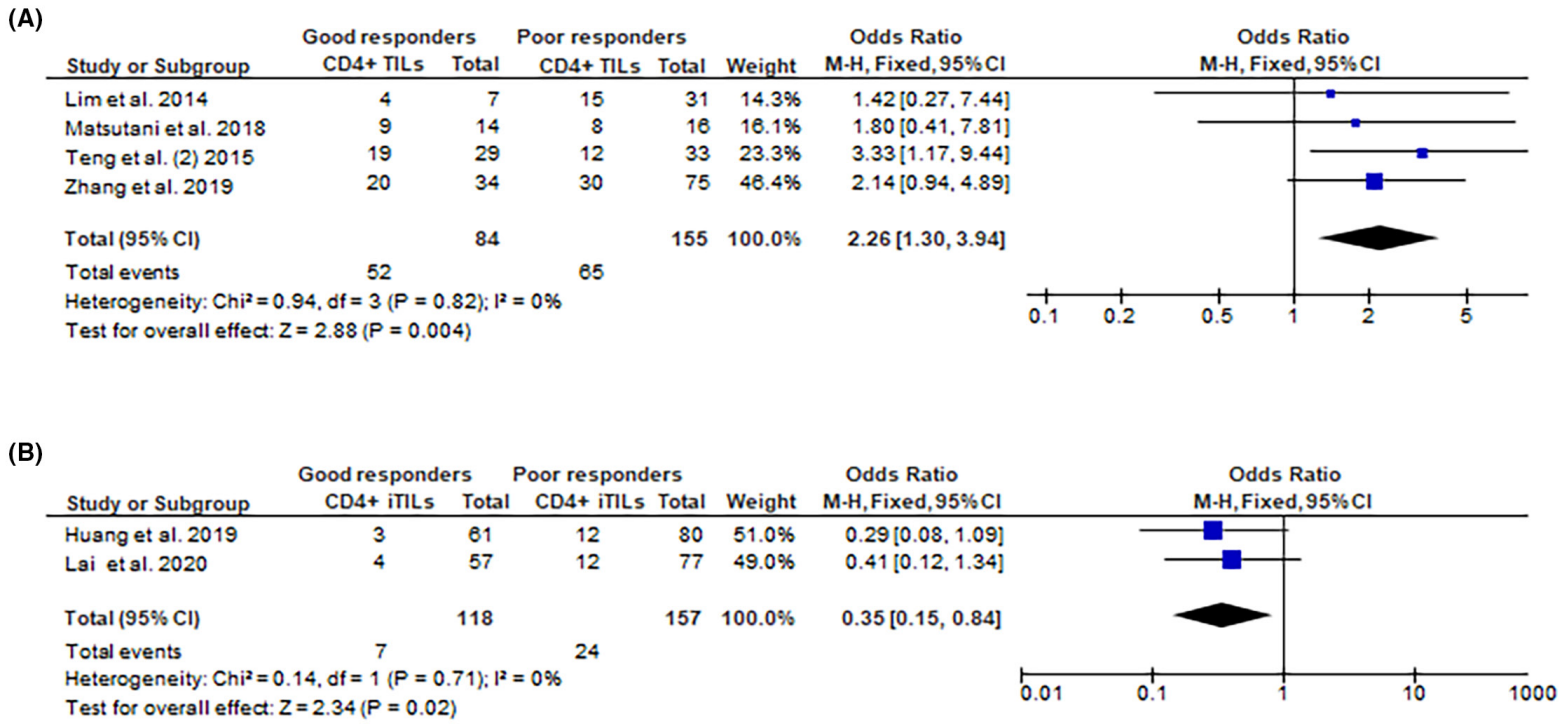
Forest plots representing the correlation between response to NT and high level of (A) total CD4+ TILs, (B) CD4+ intraepithelial TILs.

T helper (Th) cells subset is characterized by the expression of cell surface marker CD4. They are highly heterogeneous and dynamic, and they can enhance the tumor‐killing capacity of cytotoxic cells. The relationship between the presence of CD4+ Th lymphocytes and response to NT was extracted from four studies for total CD4+ TILs^16^, ^18^, ^28^, ^30^ and two studies for both CD4+ intraepithelial TILs (iTILs) and stromal TILs (sTILs)^15^, ^19^ (Figure 3). The meta‐analysis revealed a significant association between high levels of total CD4+ TILs of pretreatment biopsy specimens and good pathologic response (OR 2.26, 95% CI 1.30–3.94; participants = 239; I^2^ = 0%) (Figure 3A). On the contrary, high levels of CD4+ iTILs were associated with poor response (OR 0.35, 95% CI 0.15–0.84; participants = 275; I^2^ = 0%) (Figure 3B). We failed to find any significant evidence demonstrating the association between CD4+ sTILs of pretreatment biopsy specimens and treatment response (OR 1.36, 95% CI 0.85–2.20; participants = 278; I^2^ = 0%) (Figure S1A).

#### 3.3.3 CD8+ T lymphocyte subset

**FIGURE 4:**
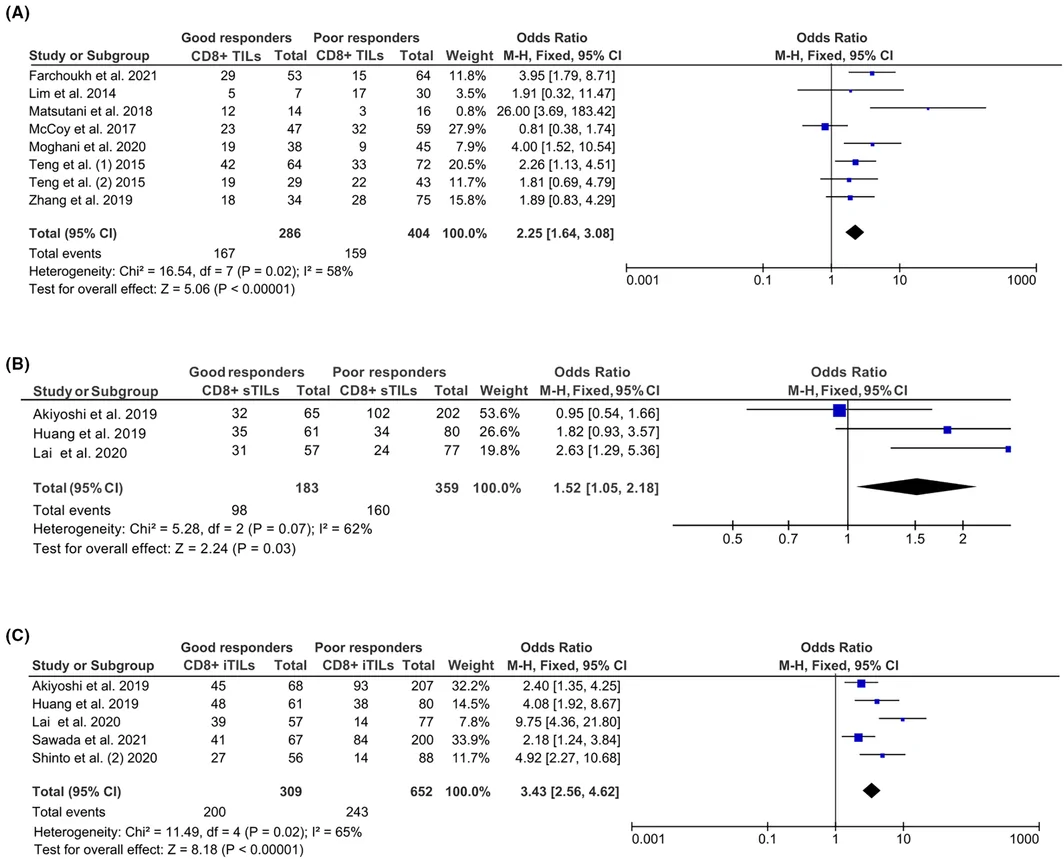
Forest plots representing the correlation between response to NT and high level of (A) total CD8+ TILs, (B) CD8+ stromal TILs, (C) CD8+ intraepithelial TILs.

CD8+ T cells are cytotoxic lymphocytes with potential tumor cell‐killing ability. The association between the presence of CD8+ cells and response to NT was extracted from eight studies for total CD8+ TILs,^16^, ^17^, ^18^, ^28^, ^29^, ^30^, ^31^, ^32^ five studies for CD8+ iTILs^15^, ^19^, ^33^, ^34^, ^35^ and three studies for CD8+ sTILs^15^, ^19^, ^32^ (Figure 4). Patients that in pretreatment biopsy specimens had high levels of total CD8+ TILs (OR 2.25, 95% CI 1.64–3.08; participants = 690; I^2^ = 58%, Figure 4A), CD8+ sTILs (OR 1.52, 95% CI 1.05–2.18; participants = 542; I^2^ = 62%, Figure 4B), as well as CD8+ iTILs (OR 3.43, 95% CI 2.56–4.62; participants = 961; I^2^ = 65%, Figure 4C), showed a significant correlation with good treatment response.

#### 3.3.4 FOXP3+ lymphocyte subset

FOXP3 is a transcription factor commonly expressed by Treg, which mediate immune tolerance. We identified four studies providing data on association between treatment response and FOXP3 expression.^16^, ^18^, ^29^, ^30^ No significant correlation was found between treatment response and FOXP3 expression (OR 0.70, 95% CI 0.44–1.12; participants = 307; I^2^ = 80%) (Figure S1B).

### 3.4 Immune checkpoint molecules as predictors of response to therapy

#### 3.4.1 PD‐L1

**FIGURE 5:**
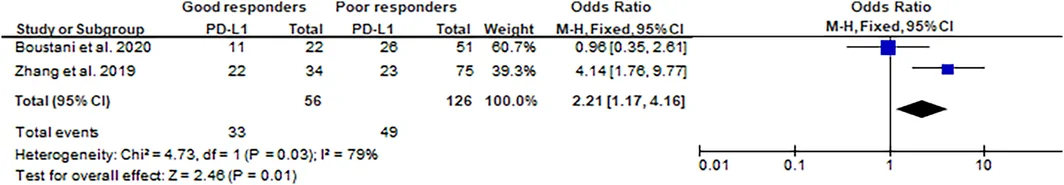
Forest plots representing the correlation between response to NT and high level of unfractionated PD‐L1.

PD‐L1 is the primary ligand of Programmed Death‐1 (PD‐1) and both tumor and immune cells can express it. Engagement of PD‐L1 with its receptor on T cells delivers an inhibitory signal. Two studies demonstrating the relationship between response to therapy and unfractionated (immune and tumor cells) PD‐L1 expression^18^, ^36^ and three studies for fractionated tumoral PD‐L1 expression in pretreatment biopsy specimens^37^, ^38^, ^39^ were included in the meta‐analysis. The results showed that high levels of unfractionated PD‐L1 are associated with a good response to NT (OR 2.21, 95% CI 1.17–4.16; patients = 182; I^2^ = 79%) (Figure 5). However, no significant correlation was observed between tumoral PD‐L1 and treatment response (OR 1.27, 95% CI 0.80–2.02; participants = 336; I^2^ = 0%) (Figure S1C).

#### 3.4.2 CTLA‐4

Cytotoxic T‐lymphocyte‐associated protein 4 (CTLA‐4) is a protein receptor that functions as an immune checkpoint downregulating immune responses. The association between the presence of CTLA4+ cells and response to NT was extracted from two studies.^18^, ^30^ No significant correlation was found between treatment response and CTLA‐4 expression in pretreatment biopsies (OR 1.00, 95% CI 0.53–1.90; participants = 171; I^2^ = 0%) (Figure S1D).

### 3.5 Publication bias

Funnel plots were generated to observe the publication bias in the meta‐analysis (Figure S2). No publication bias was observed in studies included in the analysis for the CD3+ TILs, CD4+ TILs and its intraepithelial compartment (I^2^ = 0%; *p* = 0.52; *p* = 0.82; *p* = 0.71, respectively). We found evidence of relatively high heterogeneity in studies included for the meta‐analysis for unfractionated PD‐L1, CD8 TILs, as well as its stromal and intraepithelial compartments I^2^ = 79% (*p* = 0.03); I^2^ = 58% (*p* = 0.02); I^2^ = 62% (*p* = 0.07); I^2^ = 65% (*p* = 0.02), respectively.

## 4 DISCUSSION

In this systematic review and quantitative meta‐analysis, we evaluated the predictive impact of TILs and immune checkpoint molecules in pretreatment biopsies of RC patients undergoing NT and surgery. We identified 17 studies published between November 2014 and October 2021 (representing a total of 2100 RC patients undergoing NT and with available pretreatment samples) that assessed immune biomarkers by IHC and reported TRG. Our analysis showed that patients with high pretherapeutic CD3+, CD4+ or CD8+ TILs density as well as unfractionated PD‐L1 had an augmented likelihood of achieving a good pathological response after NT. In general, this is in line with the concept that the high density of a coordinated local inflammatory infiltrate is a favorable prognostic indicator, whereas lack of it results in a poor outcome.^40^

Anitei et al. demonstrated high infiltration of CD3+ and CD8+ TILs predominated in good responders suggesting that these biomarkers could be used to predict the response to NT.^20^ Similarly, a recent study demonstrated the same pattern, adapted immunoscore of combined CD3+, and CD8+ TIL densities that was shown to be high in patients with good response.^41^ Consistent with these results, our meta‐analysis of pooled data from three studies demonstrated that high level of CD3+ TILs density could serve as a predictive biomarker of good response to NT. Moreover, our findings are consistent with the previous meta‐analysis demonstrating improved survival both for colorectal cancer patients^25^, ^42^ and improved survival and better TRG in RC patients with high density CD8+ TILs.^43^

Interestingly, our analysis demonstrated that unlike the high density of total CD4+ TIL, that is associated with good response, the high intraepithelial compartment of CD4+ TILs is associated with poor response. This might be explained by the two opposing capacities of CD4+ T cells, pro‐ and anti‐ tumorigenic.^44^ Recently, a particular attention was given to a subset of CD4+ T cells that co‐express FoxP3, Tregs.^45^ In our meta‐analysis, we evaluated four studies assessing total FoxP3 as a predictive marker of response to NT, however no significant correlation was found. Hu et al.^46^ suggested the possibility of the distinct role of FoxP3+ Tregs, which depends on their site of infiltration, namely intraepithelial or stromal locations in colorectal cancer. The stromal FoxP3+ Tregs may hinder inflammatory anti‐microbial activity allowing progression of the tumor, while intraepithelial FoxP3+ Tregs may inhibit, possibly directly contacting with tumor cells, antitumoral immune activity.^46^ Therefore, further studies investigating the density and location of Treg should be conducted for a better assessment of it as a predictive biomarker. Moreover, we cannot exclude that CD4+ cells are not only lymphocytes but also cells of the myeloid lineage.^47^ Indeed, co‐expression studies with multiplex IHC could better characterize the immune subpopulations involved in response to NT.

Pooled analysis from two studies suggested the association between high expression of unfractionated PD‐L1 and good response to NT. However, multiple studies have shown controversial results^44^: PD‐L1 appears to be induced by chemoradiotherapy, but how to interpret the levels of PD‐L1 staining before and after treatment for prediction and prognostication is still unclear.^45^ To better understand the role of PD‐L1 in the response to NT, more studies on pretreatment biopsies reporting results with distinct localization of tumoral and immune cell PD‐L1 expression are required.

Our meta‐analysis presents some limitations. First, the majority of the included studies are retrospective in nature and with small cohorts, therefore the results for some markers are based on small sample sizes. For some markers, the limited number of studies available prevented a proper assessment of the risk of publication bias. Second, different cutoff values were used to categorize patients into high‐ or low‐density groups of biomarkers in the included studies, which may explain the high heterogeneity in several results of meta‐analysis. Third, there is lack of information about the site of infiltration of TILs and the time interval between end of therapy and surgery in several studies, which might have a major effect on NT response. Moreover, some studies reported cell type variability regarding the biomarker expression. For instance, Teng et al. reported cell surface PD‐L1 expression and CTLA‐4 expression not only by tumor cells and TILs, but also histocytes.^30^ Boustani et al. evaluated unfractionated PD‐L1 expression in total tumor and stromal cells.^36^ The latter may include not only TILs, but also cells that have mesenchymal origin.^48^ Finally, we pooled data from studies using diverse NT regimens, TRG systems and different methods for biomarkers assessment (digital or manual), with some studies not clarifying the number and experience of the pathologists performing the manual assessment and patient NT therapy assignment. This might lead to imprecisions of the results due to the minor differences between TRG systems and inter‐individual variability in markers evaluation, as well as to impossibility of correlating distinct NT regimens to the response noted with specific biomarkers.

On the other hand, some important advantages of our meta‐analysis should also be addressed. It assessed distinctly different TILs subsets according to their location (intraepithelial or stromal) and immune markers according to their cellular expression. This determines a more accurate outcome for the association of biomarkers and treatment response. Moreover, to our knowledge, this is the first meta‐analysis evaluating not only TILs fractions but also immune checkpoint molecules as tissue‐based predictive biomarkers of response to NT in RC patients.

**FIGURE 6:**
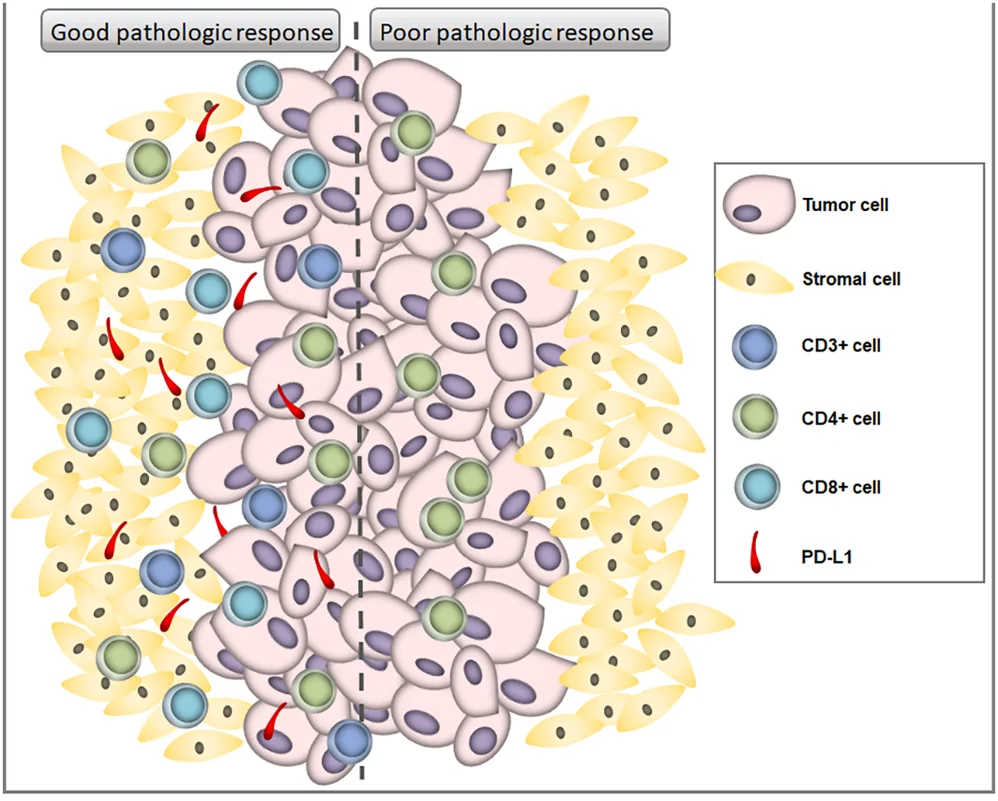
Immune markers in rectal tumor patients receiving neoadjuvant therapy associated with good or poor response to therapy.

In conclusion, our results show that high level of unfractionated CD8+ TILs together with its intraepithelial and stromal compartments, total CD4+ TILs and total CD3+ TILs may serve as predictive biomarkers of treatment response in RC patients (Figure 6). Results on unfractionated PD‐L1 expression are promising but based on limited data. These findings indicate that examining the tumoral immune infiltrate and the composition of TME prior to treatment with NT have the potential to further assist clinicians in the selection of patients that may benefit from this treatment and immune‐enhancing regimens. Larger prospective validation cohorts are warranted before these biomarkers can be used as a clinical decision marker.

## AUTHOR CONTRIBUTIONS

**Astghik Stepanyan:** Data curation (equal); formal analysis (equal); writing – original draft (equal). **Matteo Fassan:** Writing – review and editing (equal). **Gaya Spolverato:** Writing – review and editing (equal). **Ignazio Castagliuolo:** Writing – original draft (equal). **Melania Scarpa:** Conceptualization (equal); data curation (equal); writing – review and editing (equal). **Marco Scarpa:** Conceptualization (equal); supervision (equal); writing – review and editing (equal).

## FUNDING INFORMATION

The research leading to these results has received funding from AIRC under IG 2019—ID. 23381 project—P.I. Scarpa Marco.

## CONFLICT OF INTEREST STATEMENT

The authors have no conflicts of interest.

## COLLABORATORS' LIST

*IMMUNOREACT Study Group*: Agostini Marco, PhD^1,3^; Angriman Imerio, MD^1^; Bao Riccardo Quoc, MD^1^; Bardini Romeo, MD^1^; Becherucci Giulia, MD^1^; Bergamo Francesca, MD^2^; Bordignon Giovanni, MD^5^; Brignola Stefano, MD^4^; Brolese Marco, MD^1^; Businello Gianluca, MD^3^; Buzzi Gianluca, MD^9^; Campi Michela, MD^1^; Candioli Salvatore, MD^11^; Capelli Giulia, MD^3^; Cataldo Ivana, MD^4^; Cavallin Francesco, MSc^14^, Cipollari Chiara, MD^7^; Chiminazzo Valentina, MSc^1^; Da Lio Corrado, MD5; Dal Santo Luca, MD^1^; D'Angelo Antonella, MD^1^; De Simoni Ottavia, MD^2^; Dei Tos Angelo Paolo, MD^1^; Di Camillo Barbara, PhD^3^; Di Cristofaro Loretta, MD^10^; Facci Luca, MD^1^; Franzato Boris, MD^2^; Gavagna Laura, MD^11^; Godina Mario, MD^5^; Guerrieri Mario, MD^12^; Guerriero Silvio, MD^6^; Guzzardo Vincenza, MSc^3^; Kotsafti Andromachi, PhD^2^; Laurino Licia, MD^5^, Marchegiani Francesco, MD^1^; Maretto Isacco, MD^1^; Massani Marco, MD^4^; Merenda Roberto, MD^5^; Mondi Isabella, MD^5^; Negro Silvia, MD^1^; Ortenzi Monica, MD^12^; Parini Dario, MD^9^; Pilati Pierluigi, MD^2^; Pirozzolo Giovanni, MD^5^; Porzionato Andrea, MD^3^; Portale Giuseppe, MD^7^; Pozza Anna, MD^4^; Pozza Giulia, MD^1^; Prando Daniela, MD^9^; Pucciarelli Salvatore, MD^1^; Recordare Alfonso, MD^5^; Ricagna Fabio, MD^11^; Rivella Giorgio, MD^1^; Romiti Chiara, MD^6^; Ruffolo Cesare, MD^1^; Saadeh Luca, MD^1^; Salmaso Beatrice, MD^9^; Salmaso Roberta, MSc^3^; Scapinello Antonio, MD^2^; Scognamiglio Federico, BSc^1^; Spolverato Ylenia Camilla, MD^7^; Stecca Tommaso, MD^4^; Tagliente Giovanni, MD^13^; Tomassi Monica, MD^13^; Tedeschi Umberto, MD^13^; Vignotto Chiara, MD^1^; Verdi Daunia, MD^5^; Zagonel Vittorina, MD^2^; Zizzo Maurizio, MD^8^;

## COLLABORATORS' INSTITUTIONS

^1^Azienda Ospedale Università di Padova, Padova, Italy; ^2^ Veneto Institute of Oncology IOV‐IRCCS, Padova, Italy; ^3^ University of Padova, Padova, Italy; ^4^ Azienda ULSS 2 Marca Trevigiana, Treviso, Italy; ^5^ Azienda ULSS 3 Serenissima, Venezia, Italy; ^6^ Area Vasta 4 di Fermo, Ospedale Murri, Fermo, Italy; ^7^Azienda Unità Socio‐Sanitaria Locale 6 Euganea, Padova, Italy; ^8^ Azienda Unità Sanitaria Locale ‐ IRCCS di Reggio Emilia, Italy; ^9^ Azienda Unità Socio‐Sanitaria Locale 5 Polesana, Rovigo, Italy; ^10^ Ospedale GB Grassi ASL ROMA 3, Ostia, Italy; ^11^ Azienda Unità Socio‐Sanitaria Locale 1 Dolomiti, Belluno, Italy; ^12^ Azienda Ospedaliero Universitaria delle Marche, Ancona, Italy; ^13^ Policlinico Abano, Abano, Italy; ^14^ Independent Statistician, Solagna, Italy.

## Supporting information


Supplementary Figure 1

Supplementary Figure 2

**Supplementary Table S1.** TRG systems with the corresponding definitions of poor and good responders. CAP TRS=College of American Pathologists Tumor Regression Score; AJCC7/UICC, 7th American Joint Committee on Cancer/Union for International Cancer Control; JCCC 8, Japanese Classification of Colorectal Carcinoma 8th edition.

**Supplementary Table S2.** Detailed characteristics of the selected studies

**Supplementary Table S3.** Quality assessment of studies included in the meta‐analysis

## Data Availability

Data used for this systematic review and meta‐analyses were extracted from articles which were all public and available from PubMed, Ovid MEDLINE and EMBASE.
