# Correction to “IMMUNOREACT 0: Biopsy‐Based Immune Biomarkers as Predictors of Response to Neoadjuvant Therapy for Rectal Cancer—A Systematic Review and Meta‐Analysis”

**DOI:** 10.1002/cam4.72189

**Published:** 2026-08-17

A. Stepanyan , M. Fassan , G. Spolverato , I. Castagliuolo , M. Scarpa , and M. Scarpa , “IMMUNOREACT 0: Biopsy‐Based Immune Biomarkers as Predictors of Response to Neoadjuvant Therapy for Rectal Cancer**—**A Systematic Review and Meta‐Analysis,” Cancer Medicine 12 (2023): 17878–17890, 10.1002/cam4.6423.37537787 PMC10523971

The first and last names of the members of the IMMUNOREACT Study Group were swapped and have been updated in the Collaborators' List. The updated Collaborators' List is as follows:

**Collaborators' List**

*IMMUNOREACT Study Group*: Marco Agostini, PhD^1^˒^3^; Imerio Angriman, MD^1^; Quoc Riccardo Bao, MD^1^; Romeo Bardini, MD^1^; Giulia Becherucci, MD^1^; Francesca Bergamo, MD^2^; Giovanni Bordignon, MD^5^; Stefano Brignola, MD^4^; Marco Brolese, MD^1^; Gianluca Businello, MD^3^; Gianluca Buzzi, MD^9^; Michela Campi, MD^1^; Salvatore Candioli, MD^11^; Giulia Capelli, MD^3^; Ivana Cataldo, MD^4^; Francesco Cavallin, MSc^14^; Chiara Cipollari, MD^7^; Valentina Chiminazzo, MSc^1^; Corrado Da Lio, MD^5^; Luca Dal Santo, MD^1^; Antonella D'Angelo, MD^1^; Ottavia De Simoni, MD^2^; Angelo Paolo Dei Tos, MD^1^; Barbara Di Camillo, PhD^3^; Di Loretta Cristofaro, MD^10^; Luca Facci, MD^1^; Boris Franzato, MD^2^; Laura Gavagna, MD^11^; Mario Godina, MD^5^; Mario Guerrieri, MD^12^; Silvio Guerriero, MD^6^; Vincenza Guzzardo, MSc^3^; Andromachi Kotsafti, PhD^2^; Licia Laurino, MD^5^; Francesco Marchegiani, MD^1^; Isacco Maretto, MD^1^; Marco Massani, MD^4^; Roberto Merenda, MD^5^; Isabella Mondi, MD^5^; Silvia Negro, MD^1^; Monica Ortenzi, MD^12^; Dario Parini, MD^9^; Pierluigi Pilati, MD^2^; Giovanni Pirozzolo, MD^5^; Andrea Porzionato, MD^3^; Giuseppe Portale, MD^7^; Anna Pozza, MD^4^; Giulia Pozza, MD^1^; Daniela Prando, MD^9^; Salvatore Pucciarelli, MD^1^; Alfonso Recordare, MD^5^; Fabio Ricagna, MD^11^; Giorgio Rivella, MD^1^; Chiara Romiti, MD^6^; Cesare Ruffolo, MD^1^; Luca Saadeh, MD^1^; Beatrice Salmaso, MD^9^; Roberta Salmaso, MSc^3^; Antonio Scapinello, MD^2^; Federico Scognamiglio, BSc^1^; Ylenia Camilla Spolverato, MD^7^; Tommaso Stecca, MD^4^; Giovanni Tagliente, MD^13^; Monica Tomassi, MD^13^; Umberto Tedeschi, MD^13^; Chiara Vignotto, MD^1^; Daunia Verdi, MD^5^; Vittorina Zagonel, MD^2^; Maurizio Zizzo, MD^8^.

The online version of this article has been corrected accordingly.

We apologize for this error.

