# Supplementary figures and images for: IMMUNOREACT 0: Biopsy‐based immune biomarkers as predictors of response to neoadjuvant therapy for rectal cancer—A systematic review and meta‐analysis
Source: Cancer Med. 2023 Aug 3;12(17):17878–90. doi: 10.1002/cam4.6423 (PMC10523971; doi:10.1002/cam4.6423)

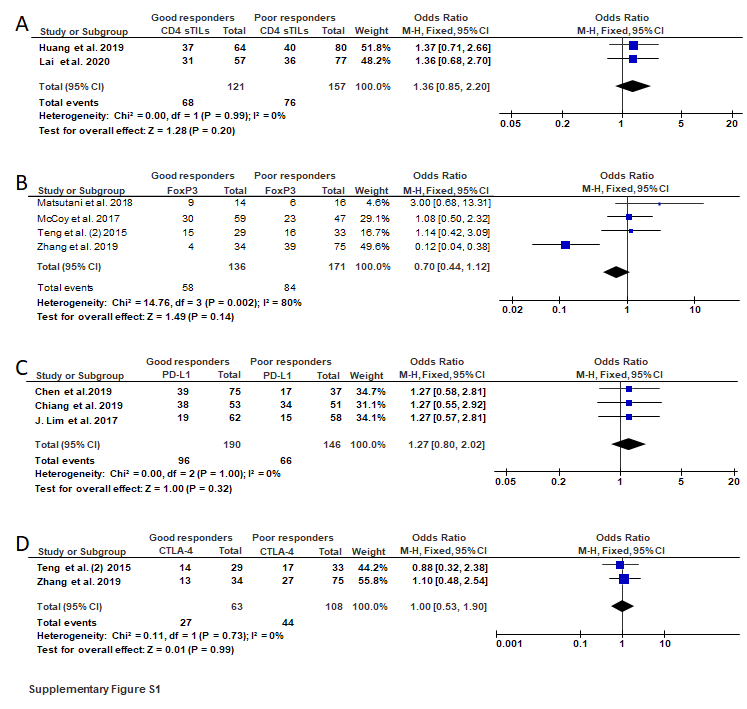

Supplement: Supplementary file 1 — Supplementary Figure 1 [file CAM4-12-17878-s001.tiff]

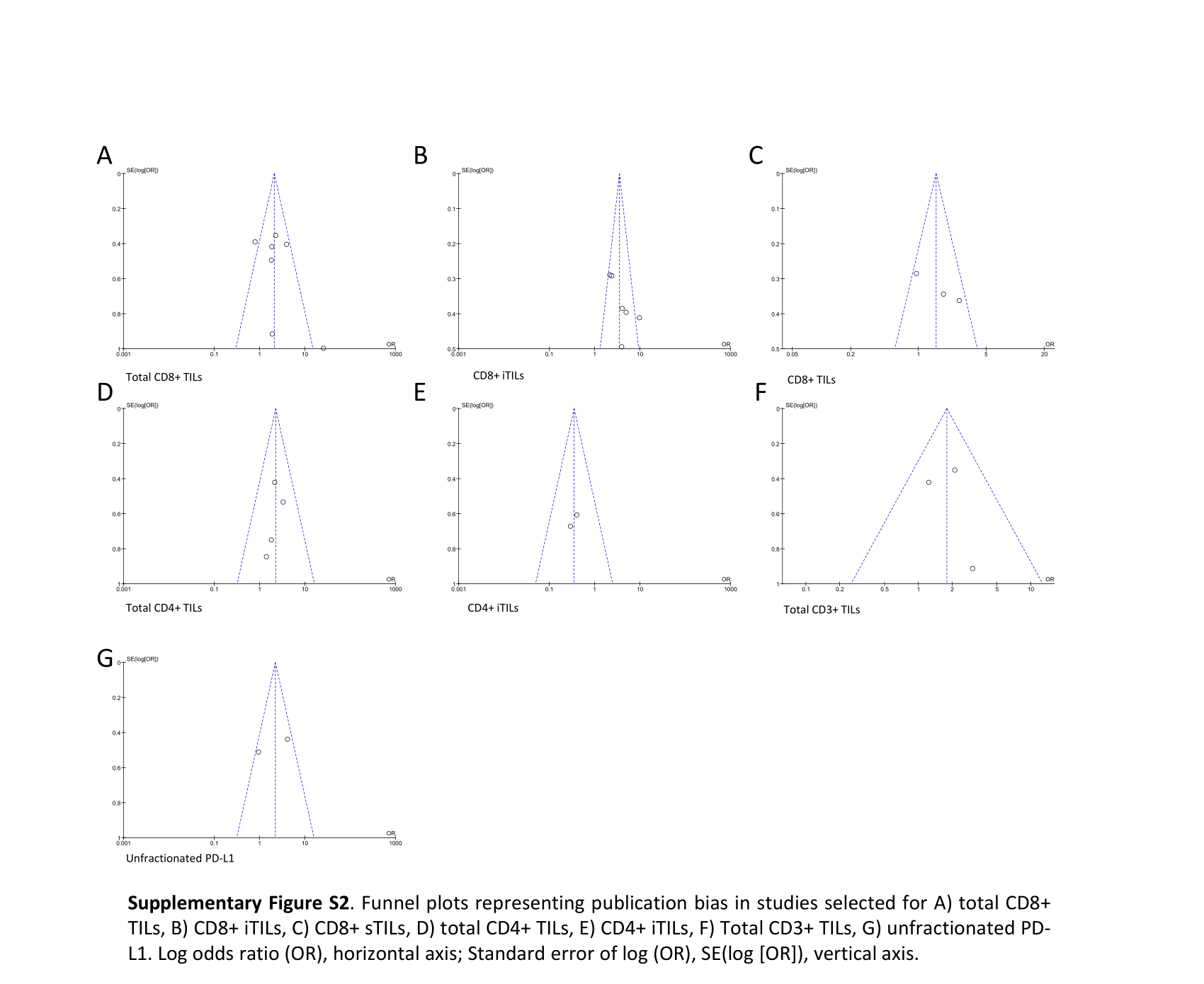

Supplement: Supplementary file 2 — Supplementary Figure 2 [file CAM4-12-17878-s004.tiff]
