# Supplementary material for: IMMUNOREACT 0: Biopsy‐based immune biomarkers as predictors of response to neoadjuvant therapy for rectal cancer—A systematic review and meta‐analysis
Source: Cancer Med. 2023 Aug 3;12(17):17878–90. doi: 10.1002/cam4.6423 (PMC10523971; doi:10.1002/cam4.6423)
Supplement: Supplementary file 3 — Supplementary Table S1. TRG systems with the corresponding definitions of poor and good responders. CAP TRS=College of American Pathologists Tumor Regression Score; AJCC7/UICC, 7th American Joint Committee on Cancer/Union for International Cancer Control; JCCC 8, Japanese Classification of Colorectal Carcinoma 8th edition. [file CAM4-12-17878-s005.docx]

**Supplementary Table S1.** **TRG systems with the corresponding definitions of poor and good responders.** CAP TRS=College of American Pathologists Tumor Regression Score; AJCC7/UICC=7^th^ American Joint Committee on Cancer/Union for International Cancer Control; JCCC 8=Japanese Classification of Colorectal Carcinoma 8^th^ edition.

| **TRG system** | **Good responders** | **Poor responders** |
| --- | --- | --- |
| **Dworak’s TRG** | 3-4 | 1-2 |
| **Mandard’s TRG** | 1-2 | 3-5 |
| **CAP TRS** | 2-3 | 0-1 |
| **AJCC 7/UICC** | 0-1 or complete-moderate | 2-3 or minimal-poor |
| **JCCC8** | 1b-3 | 0-1a |
